# Supplementary material for: Single-cell profiling of Anopheles gambiae spermatogenesis defines the onset of meiotic silencing and premeiotic overexpression of the X chromosome
Source: Commun Biol. 2023 Aug 15;6:850. doi: 10.1038/s42003-023-05224-z (PMC10427639; doi:10.1038/s42003-023-05224-z)
Supplement: Supplementary file 2 — Supplementary Information [file 42003_2023_5224_MOESM2_ESM.pdf]

Supplementary Material

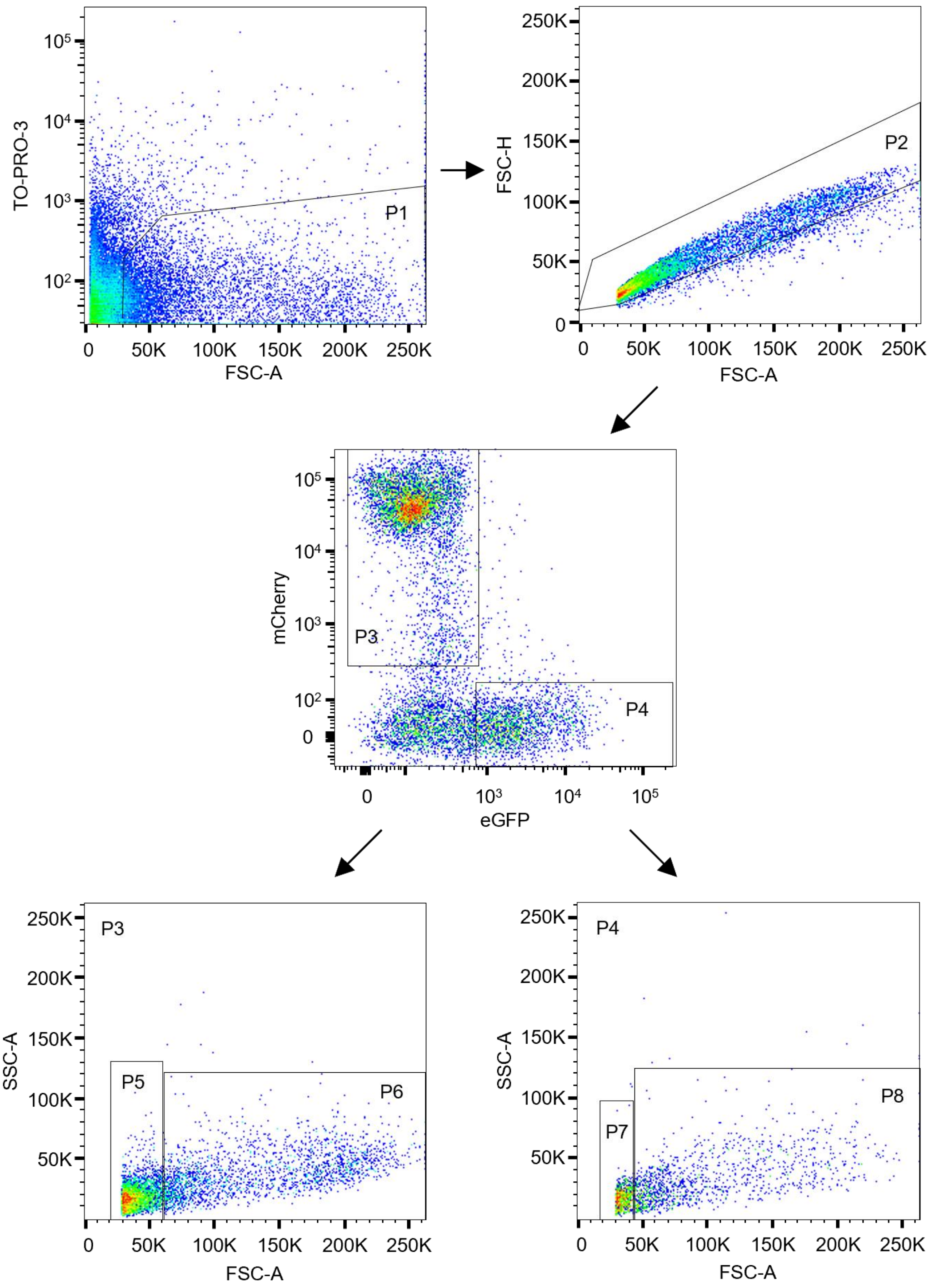

Supplementary Figure 1: Fluorescent activated cell sorting of testicular germline cell suspensions. *Flow cytometric isolation of cells for single cell sequencing, presented as pseudocolour FlowJo dot plots, based on TO-PRO-3 iodide and FSC-A (P1) to exclude dead cells and small sized debris, FSC-H and FSC-A to include only singlets (P2) and mCherry and eGFP fluorescence intensity of live cells to include only cells positive for either mCherry (P3) or eGFP (P4) fluorescence. Positive for mCherry (P3) or eGFP (P4) cells were additionally gated for cell size based on FSC-A and SSC-A and sorted into two populations each, representing smaller (P5 or P7, respectively) and larger cells (P6 or P8, respectively).*

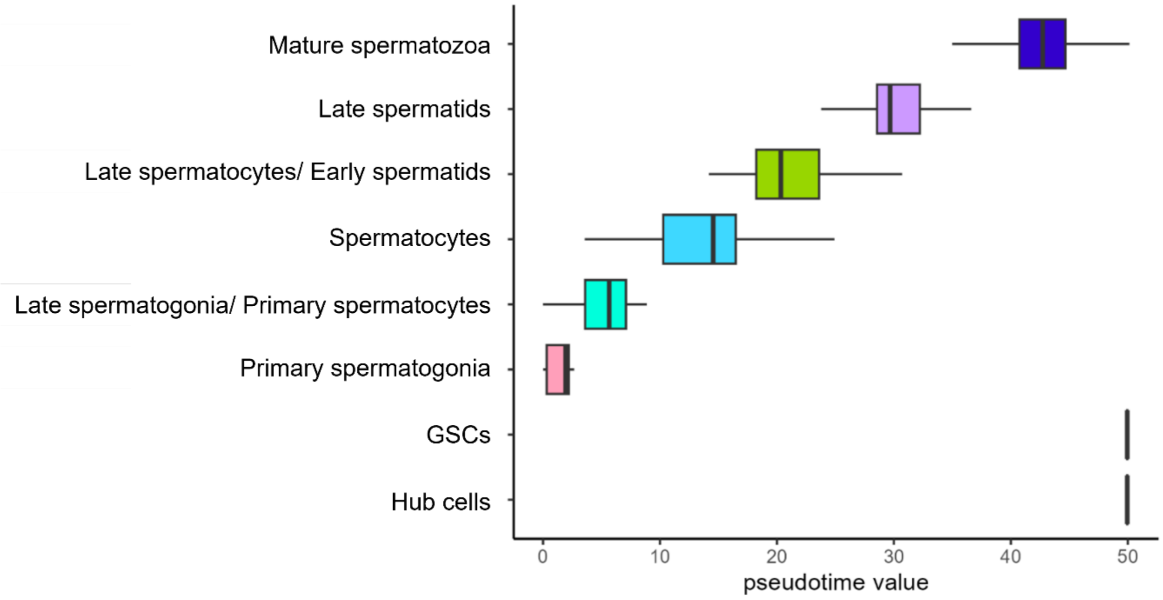

Supplementary Figure 2: Pseudotime values per cell cluster. *Box and whisker plots showing the pseudotime values assigned to cells within each cell type cluster. The pseudotime start point was assigned to the early stages of spermatogenesis based on known expression of the vasa (AGAP008578) gene. Higher pseudotime values indicate further differentiation from the start of spermatogenesis. The lower and upper quartiles are defined by the box with the median represented within. Whiskers show the maximum and minimum range of the data.*

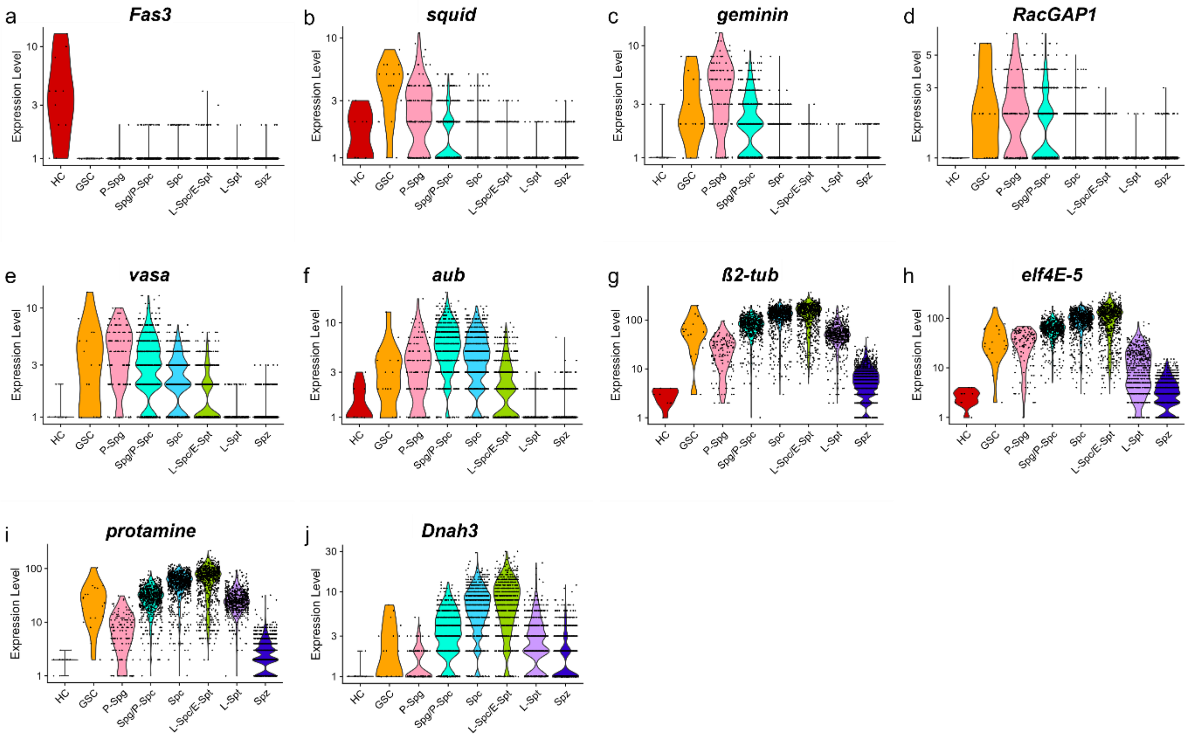

Supplementary Figure 3: Expression profiles levels of genes used in cell type assignment. *Genes include (a) Fas3 (AGAP029564), (b) squid (AGAP000399), (c) geminin (AGAP000496), (d) RacGAP1 (AGAP008912), (e) vasa (AGAP008578), (f) aubergine (AGAP011204), (g) ß2-tubulin (AGAP008622), (h) protamine (AGAP028569) and (i) Dnah3 (AGAP007675). Dots show the log-transformed expression values for each cell within the cluster; Hub cells (HC), Germline stem cells (GSC), Primary spermatogonia (P-Spg), Late Spermatogonia/Primary spermatocytes (L- Spg/P-Spc), Spermatocytes (Spc), Late spermatocytes/Early spermatids (L-Spc/E-Spt), Late spermatids (L-Spt) and Mature spermatozoa (Spz).*

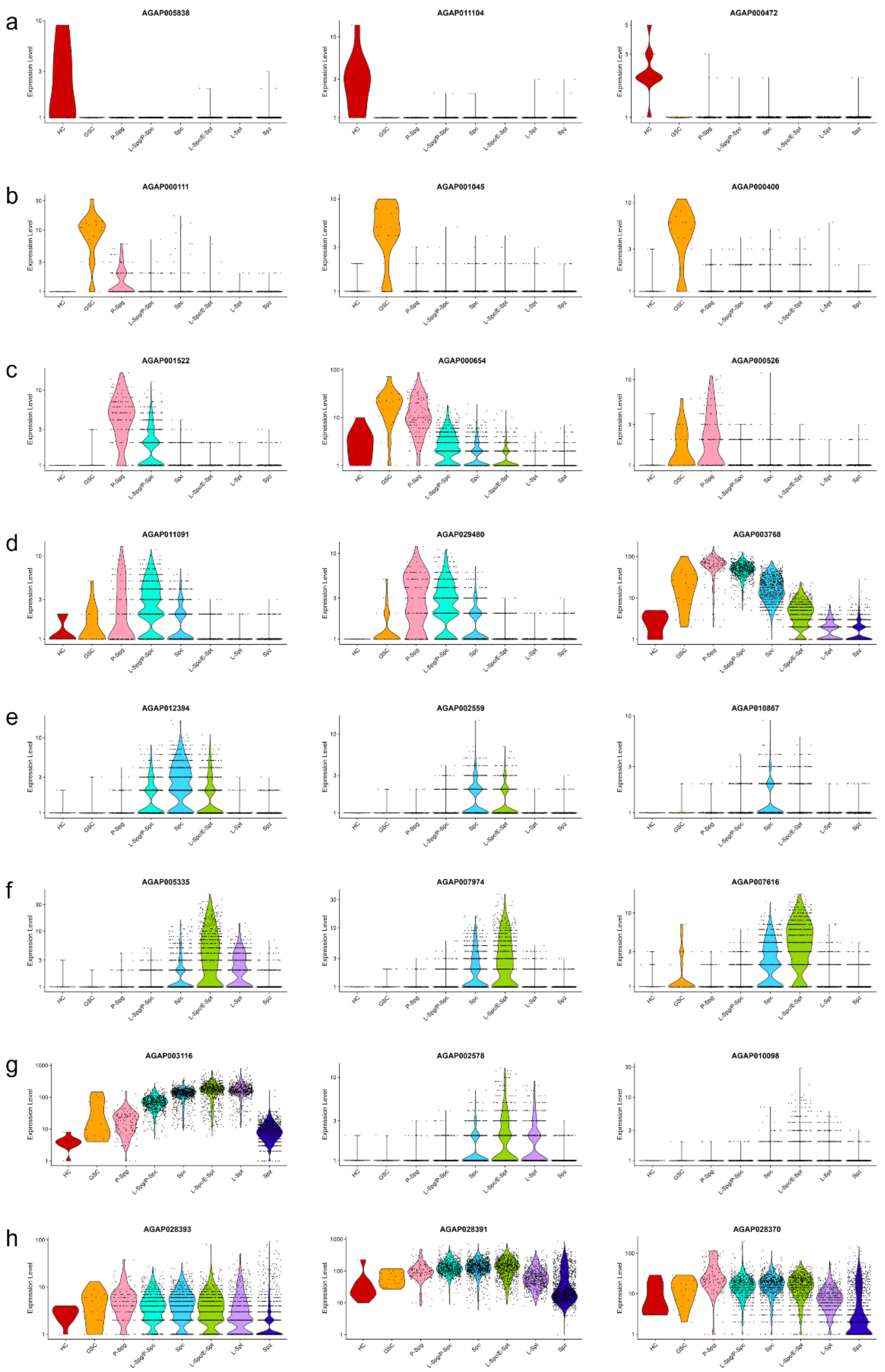

Supplementary Figure 4: Expression profiles of top 3 enriched genes per cell cluster. *Dots show the log-transformed expression values for each cell within the cluster. Expression profiles shown for the top 3 most enriched genes in Hub cells (HC)(a), Germline stem cells (GSC)(b), Primary spermatogonia (P-Spg)(c), Late Spermatogonia/Primary spermatocytes (L-Spg/P-Spc)(d), Spermatocytes (Spc)(e), Late spermatocytes/Early spermatids (L-Spc/E-Spt)(f), Late spermatids (L-Spt)(g) and Mature spermatozoa (Spz)(h). These genes were selected from the significantly enriched genes found in the differential expression analysis (Table S1) and the 3 most significant (lowest p-value) were isolated for each cell type cluster.*

Cell cluster	*X*	*Autosome*	*X/A*	*p-value*	*Adjusted p-value*
Hub cells	0.000	0.000	1.000	9.74E-01	1.00E+00
GSCs	2.585	2.322	1.113	2.08E-04	1.66E-03
Primary spermatogonia	2.807	5.755	0.488	2.62E-72	2.10E-71
Spermatogonia/ Primary spermatocytes	2.585	8.414	0.307	4.27E-182	3.42E-181
Spermatocytes	2.585	8.755	0.295	3.03E-209	2.43E-208
Late spermatocytes/ Early spermatids	2.000	7.830	0.255	1.56E-224	1.24E-223
Late spermatids	1.000	4.585	0.218	5.51E-165	4.41E-164
Mature spermatozoa	1.000	4.700	0.213	3.24E-153	2.59E-152

Supplementary Table 1: Gene expression ratios for genes located on X or Autosomal (A) chromosomes per cell cluster. *The median log transformed counts (Log2(counts +1)) per gene is indicated for each cell cluster and the X/A ratio calculated from the median values. The p-values shown are for a Wilcoxon test to determine if the X:A ratio significantly differs from the null hypothesis ratio of 1. Adjusted p-values were calculated using the "Bonferroni" correction for multiple testing.*
