# Supplementary material for: Single-cell profiling of Anopheles gambiae spermatogenesis defines the onset of meiotic silencing and premeiotic overexpression of the X chromosome
Source: Commun Biol. 2023 Aug 15;6:850. doi: 10.1038/s42003-023-05224-z (PMC10427639; doi:10.1038/s42003-023-05224-z)
Supplement: Supplementary file 3 — Description of Additional Supplementary Files [file 42003_2023_5224_MOESM3_ESM.pdf]

Description of Additional Supplementary Files

File name: Supplementary Data 1
Description: Differential expression analysis and cluster-enriched genes

File name: Supplementary Data 2
Description: Gene Ontology overrepresentation analysis

File name: Supplementary Data 3
Description: Source data for Figure 3. Feature table showing which genes are significantly enriched (1) or not enriched (0) in a specific cell-type cluster. Additional data shows total number of significantly enriched genes for in chromosome in each cell-type cluster.

File name: Supplementary Data 4
Description: Source data for Figure 4. Sum of Unique Molecular Identifiers (UMIs), alternatively referred to as transcript reads, for each cell grouped by cluster. Data shown for Autosome, X chromosome and Y contigs in separate tables.

File name: Supplementary Data 5
Description: Source data for Figure 5. Raw counts and log-transformed (log2(counts+1)) counts per cell grouped by cell cluster and chromosome.
